# Combined intracellular nitrate and NIT2 effects on storage carbohydrate metabolism in *Chlamydomonas*

**DOI:** 10.1093/jxb/ert339

**Published:** 2013-11-01

**Authors:** C. Remacle, G. Eppe, N. Coosemans, E. Fernandez, H. Vigeolas

**Affiliations:** ^1^University of Liege, Institute of Botany, B22, Genetics of Microorganisms, 4000 Liege, Belgium; ^2^University of Liege, Inorganic Analytical Chemistry, LSM-CART, Allée de la Chimie B6c, 4000 Liege, Belgium; ^3^Departamento de Bioquımica y Biologıa Molecular, Facultad de Ciencias, Universidad de Cordoba, Campus de Rabanales, 14071 Cordoba, Spain

**Keywords:** Biomass, *Chlamydomonas*, fatty acid, nitrate, nitrogen, oil, starch.

## Abstract

Microalgae are receiving increasing attention as alternative production systems for renewable energy such as biofuel. The photosynthetic alga *Chlamydomonas reinhardtii* is widely recognized as the model system to study all aspects of algal physiology, including the molecular mechanisms underlying the accumulation of starch and triacylglycerol (TAG), which are the precursors of biofuel. All of these pathways not only require a carbon (C) supply but also are strongly dependent on a source of nitrogen (N) to sustain optimal growth rate and biomass production. In order to gain a better understanding of the regulation of C and N metabolisms and the accumulation of storage carbohydrates, the effect of different N sources (NH_4_NO_3_ and ) on primary metabolism using various mutants impaired in either *NIA1*, *NIT2* or both loci was performed by metabolic analyses. The data demonstrated that, using NH_4_NO_3_, *nia1* strain displayed the most striking phenotype, including an inhibition of growth, accumulation of intracellular nitrate, and strong starch and TAG accumulation. The measurements of the different C and N intermediate levels (amino, organic, and fatty acids), together with the determination of acetate and  remaining in the medium, clearly excluded the hypothesis of a slower  and acetate assimilation in this mutant in the presence of NH_4_NO_3_. The results provide evidence of the implication of intracellular nitrate and NIT2 in the control of C partitioning into different storage carbohydrates under mixotrophic conditions in *Chlamydomonas*. The underlying mechanisms and implications for strategies to increase biomass yield and storage product composition in oleaginous algae are discussed.

## Abstract (precis)

Metabolic analysis of various mutants impaired in nitrate assimilation pathways (NIA1, NIT2 loci) revealed the essential role of NIT2 and intracellular nitrate in the control of biomass yield and storage carbohydrate product biosynthesis, such as starch, oil, precursors of biofuel

## Introduction

For photosynthetic organisms, such as higher plants and green microalgae, inorganic nitrogen (N) is not only one of the essential nutrients but also the most limiting mineral element for growth and yield (Kropat *et al.*, 2011). Ammonium (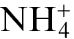) and nitrate (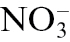) are the major primary sources of N in higher plants and microorganisms, and their respective use is strongly dependent on the species and the environmental conditions.

Assimilation of inorganic N into amino acids and proteins requires both energy and organic carbon skeletons, leading to strong interactions between N and C assimilation (Huppe *et al.*, 1994). 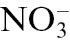 uptake and assimilation involved two ‘transport’ steps (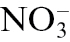 transport into the cells and nitrite transport into the chloroplasts) and two ‘reduction’ steps (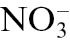. and nitrite reductases, NR and NiR, respectively), leading to 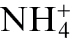, which is directly incorporated into central C metabolism. In *Chlamydomonas*, nitrate reduction is catalysed by a homodimeric NAD(P)H–NR complex containing two activities: NAD(P)H-cytochrome *c* reductase (diaphorase, EC 1.6.6.1-3) and reduced benzyl viologen NRs (terminal NR) (Kalakoutskii and Fernández, 1995).

In *Chlamydomonas*, the predominant route of 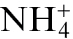 assimilation is the glutamine synthetase/glutamate synthase cycle (GS/GOGAT cycle). GS (EC 6.3.1.2) catalyses the transfer of 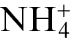 to glutamate, leading to the formation of glutamine. Subsequently, GOGAT (EC 1.4.1.4) catalyses the formation of two molecules of glutamate from one molecule of glutamine and one molecule of α-ketoglutarate. Other sources of intracellular 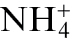 are photorespiration, protein turnover, and nucleic acid catabolism. Indeed, it has been shown that as much as 50% of C in algae is integrally coupled with N metabolism (Vanlerberghe *et al.*, 1991), suggesting that biomass composition of algae is strongly affected by variation in both C and N partitioning within cells. This C/N balance is supported by several studies in many microalgae, including *Chlamydomonas*, that demonstrate the enhancement of oil and starch accumulation under N-deficient conditions (Work *et al.*, 2010; Fan *et al.*, 2012). Unfortunately, the resulting high oil and starch contents per cell are also accompanied by a slower growth rate leading to decreased biomass productivity, which is not sustainable for commercial, biotechnological applications.

Biomass is composed mainly of proteins, carbohydrates (starch), and lipids, the proportion of each depending on the strain and culture condition (Liang *et al.*, 2009). Many microalgae are able to produce a large amount of oil, which has been widely considered as a promising source of renewable production of biodiesel to petroleum fuels (Wijffels and Barbosa, 2010). Lipids are synthesized via a complex set of pathways involving co-operation between plastidial and cytosolic metabolism. The polysaccharide starch, which is the dominant storage C product in *Chlamydomonas*, is produced within plastids. ADP-glucose pyrophosphorylase (AGPase) is the key enzyme in the regulation of starch biosynthesis in higher plants and green algae (Van den Koornhuyse *et al.*, 1996; Zabawinski *et al.*, 2001; Vigeolas *et al.*, 2004). The synthesis of fatty acids (FAs) and starch occurs in the same compartments and requires the same precursors, suggesting a competition of the two pathways for the shared substrates or at least an interaction in higher plants (Zabawinski *et al.*, 2001; Vigeolas *et al.* 2004; Li *et al.*, 2010*a*,*b*). In green algae, this notion is supported by recent data showing that a *Chlamydomonas* starchless mutant with a large decrease in AGPase activity displays TAG accumulation under specific stress conditions (Li *et al.*, 2010*a*,*b*).

In order to efficiently modulate N assimilation and its allocation, cells developed signalling mechanisms to sense N and induce gene expression. The tight control of C/N metabolism involves, besides sugar sensing and its signalling pathways, signals produced from 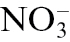, 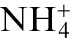, and other N metabolites such as glutamate, glutamine, and aspartate (Stitt and Krapp, 1999; Coruzzi and Zhou, 2001; Miller *et al.*, 2008). 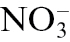, for example, is not only an essential nutrient but also a key N signalling molecule, regulating the expression of genes involved in N assimilation and primary metabolism, as well as cellular and developmental processes (Scheible *et al.*, 1997, 2004; Zhang and Forde, 1998; Wang *et al.*, 2003; Gerin *et al.*, 2010).

In plants, several potential regulatory 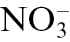 genes have been isolated and their role in 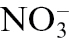 signalling has been studied (Daniel-Vedele *et al.*, 1998). For example, the *Arabidopsis* NLP7 (Nin-like protein 7) modulates 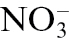 signalling and metabolism (Castaings *et al.*, 2009) and shows conservation with the *Chlamydomonas* NIT2 protein, both of which are RWP-RK transcriptional factors (Camargo *et al.*, 2007; Konishi and Yanagisawa, 2013). Hormones, such as cytokinin, that respond to the N supply, clearly interact with N regulators, and regulate metabolism and development (Coruzzi and Zhou, 2001; Sakakibara, 2006; Argueso *et al.*, 2009).

The availability of a large collection of mutants affected in most of the steps of 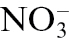 uptake and assimilation makes *Chlamydomonas* an interesting model to study N signalling in green algae. Indeed, negative (*NRG1-4*, *FAR1*, *CYG56*) and positive (*NIT2*) regulatory loci for N uptake and metabolism, which participate in N signalling have already been characterized in *Chlamydomonas* (Gonzalez-Ballester *et al.*, 2005; Fernandez and Galvan, 2007; de Montaigu *et al.*, 2010). Based on expression studies, a number of genes involved in 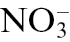. assimilation have been shown to be positively regulated by *NIT2*, such as genes involved in 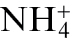 uptake repression and 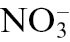 induction, including *NIA1* and *NIR1*, encoding NR and NiR, respectively (Fernandez *et al.*, 1989; Quesada *et al.*, 1998*a*) and genes involved in N transport, such as *NRT2;1*, *NRT2;2*, *NRT2;3 NAR2*, and *NAR1* encoding 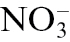/nitrite transporters (Quesada *et al.*, 1998*b*; Rexach *et al.*, 2000). Despite the role of 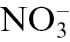 on its own metabolism, it has also been demonstrated that the latter is involved in 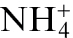 uptake and acetate assimilation by repressing *NIA1* gene expression, and inducing acetyl-CoA synthetase, respectively (Llamas *et al.*, 2002; Gerin *et al.*, 2010).

In this work, the potential regulatory effects of *NIA1* and *NIT2*, encoding NR and the regulatory protein for nitrate assimilation, respectively, on primary C metabolism was investigated in four different NR-deficient strains displaying a mutation in *NIA1*, *NIT2* (*nit2.1* and *nit2.2* strains) or both loci (Fernandez and Matagne, 1986). The study of the role of NIA1 and NIT2 in 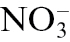 signalling and the effect on primary metabolism is partly complicated by the fact that none of these different NR-deficient mutants can grow on 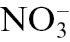 as the sole N source (Fernandez and Matagne 1986). For this purpose, all strains were grown under mixotrophic condition in the presence of either 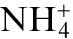 or NH_4_NO_3_. Acetate, which is rapidly incorporated into tricarboxylic acid cycle intermediates, via acetyl-CoA synthetase, required for 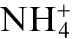 incorporation into primary C metabolism, was chosen as organic carbon source. Metabolomic approaches were employed to identify biochemical changes that may be linked directly or indirectly to these two loci. It was shown that intracellular 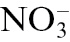 and NIT2 participate in the control of C partitioning into different C storage pools under mixotrophic conditions in *Chlamydomonas*.

## Materials and methods

### Strains and culture conditions

The wild-type 21gr strain and the mutants *nia1* (305), *nit2.1* (nit2), *nit2.2* (203) and *nia1nit2* (137c) have been characterized previously (Fernández and Matagne, 1984). Strains were grown at 25 °C in Tris-acetate-phosphate 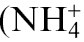 medium 7mM) or Tris-acetate-phosphate-ammonium nitrate 7mM (NH_4_NO_3_ medium) liquid or solid (1.5% agar) medium, under continuous light (50 μE m^–2^ s^−1^) as described by Harris (1989). Cell counts were assessed using a Beckman Z2 Coulter cell and particle counter (Beckman Coulter).

### Mass spectroscopy analyses of FA methyl esters (FAMEs)

Lipids were extracted and derivatized from liquid culture. Briefly, 1.0ml of methanol saturated with 1M HCl was added to 1ml of culture and heated in tightly sealed vials at 80 °C for 90min, resulting in cell lysis and lipid saponification. FAMEs were then extracted into 2ml of 1:1 hexane in 0.9% NaCl via gentle inversion. Hexane extracts, containing FAMEs, were measured using a Trace GC2000-PolarisQ ion trap mass spectrometer (Thermo-Scientific, Waltham, MA, USA) equipped with a CTC Combi-Pal autosampler (CTC Analytics, Zwingen, Switzerland), using the GC column (SP2331, 30 m×0.25mm×0.20 µm film thickness; Supelco Bellefonte USA). Pentadecanoic acid (C15:0) was also used as an internal standard for quantification.

### Thin layer chromatography (TLC) of the neutral lipid fraction

Using freeze-dried cells (50ml of algal culture), lipids were extracted according to the method of Bligh and Dyer (1959). Chloroform extracts corresponding to 10^7^ cells were fractionated by TLC, as described by Stobart *et al.* (1997). The staining of the TLC plate was done with iodine vapour.

### Determination of cellular dry weight, starch, and protein levels

Dry weight, starch, and protein contents were measured as described by Vigeolas *et al.* (2012).

### Determination of total free amino acid, nitrate, and malic and fumaric acid contents

Metabolites were extracted twice with 80% ethanol and one with 50% ethanol. Total free amino acids were assayed according to Bantan-Polak *et al.* (2001). Malate, fumarate, and nitrate levels were measured as described by Tschoep *et al.* (2009).

### Determination of 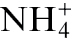 and acetate contents


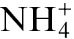
 and acetate contents were measured by using Megazyme assay K-AMIAR and K-ACETAK kits, respectively (Megazyme, Wicklow, Ireland).

### Chemicals

Unless stated otherwise, chemicals were obtained from Sigma (Taufkirchen, Germany) or Merck (Darmstadt, Germany).

## Results

### Growth rate is strongly affected in a *nia1* mutant in the presence of NH_4_NO_3_ as the N source

**Fig. 1.**
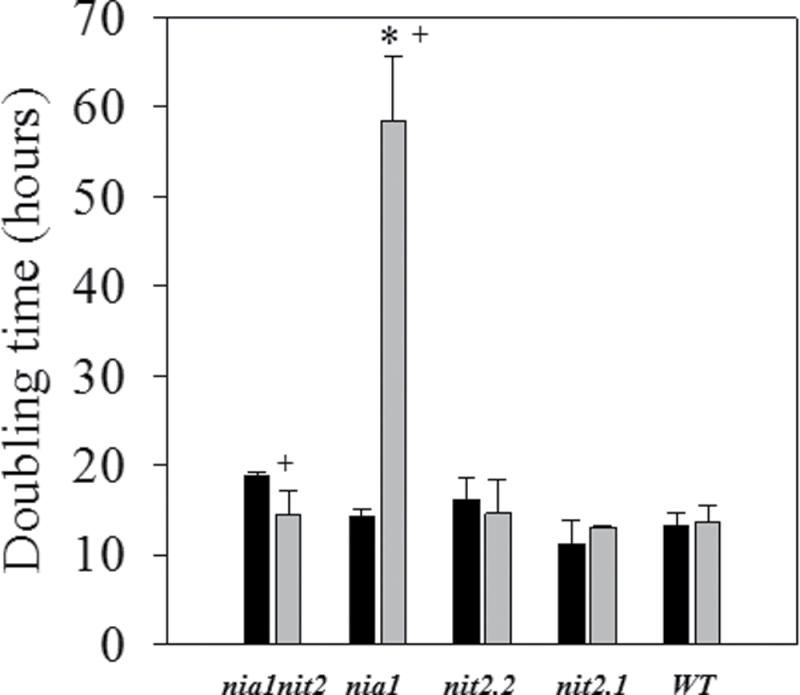
*Chlamydomonas* growth on 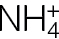 and NH_4_NO_3_. Cells of wild-type (WT) and NR-deficient strains were grown in acetate medium containing 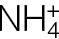 (black bars) or NH_4_NO_3_ (grey bars). Values are means±SE (*n*=3–6). Asterisks represent values significantly different from the wild type; + represents a significantly different value between 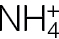 and NH_4_NO_3_ cultures for each particular strain (based on Student’s *t*-test with *P*≤0.05).

Growth rate strongly depends on the nutrient availability in the medium, with N and C being the most important macronutrients. As NR-deficient strains are not able to grow on nitrate as the sole source of N, analyses of the *nia1*, *nit2*, and *nia1nit2* strains were performed under two different mixotrophic mediums, containing either 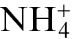 or NH_4_NO_3_. Using only 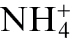 as a source of N, all the NR-deficient and wild-type strains displayed similar growth rates (Fig. 1), suggesting that 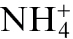 assimilation is unaffected in the mutant strains. In NH_4_NO_3_ medium, the wild-type strain, the *nia1nit2* double mutant, and both *nit2* mutant strains displayed unchanged growth rates compared with pure 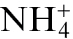 nutrition. In contrast, the *nia1* strain grew significantly slower using NH_4_NO_3_ compared with 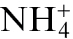 as the source of N in the medium. These values were consistent with data published previously (Fernandez and Cardenas, 1982).

### The *nia1* mutant displays stimulation of acetate and 
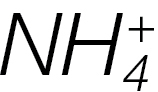
 uptake under NH_4_NO_3_

**Fig. 2.**
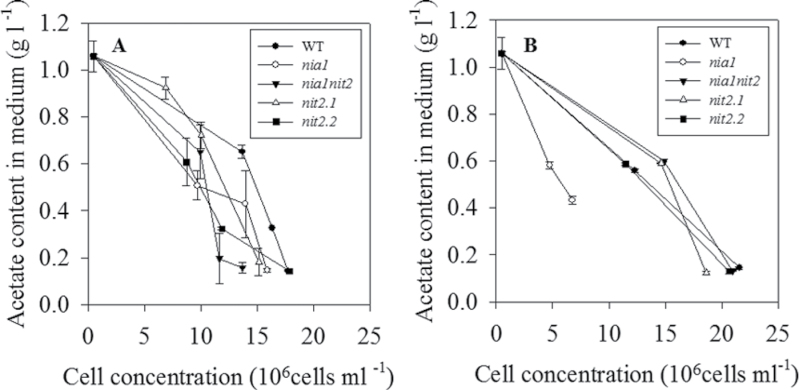
Concentrations of acetate remaining in wild-type (WT) and NR-deficient strains in acetate medium containing either 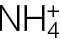 (A) or NH_4_NO_3_ (B). Algae cultures were inoculated at 5×10^5^ cells ml^–1^. The initial acetate concentration in acetate in both media was 1g l^–1^. During the exponential growth phase, the level of acetate remaining in medium was determined (see Materials and methods). Values are means±SE (*n=3*).

**Fig. 3.**
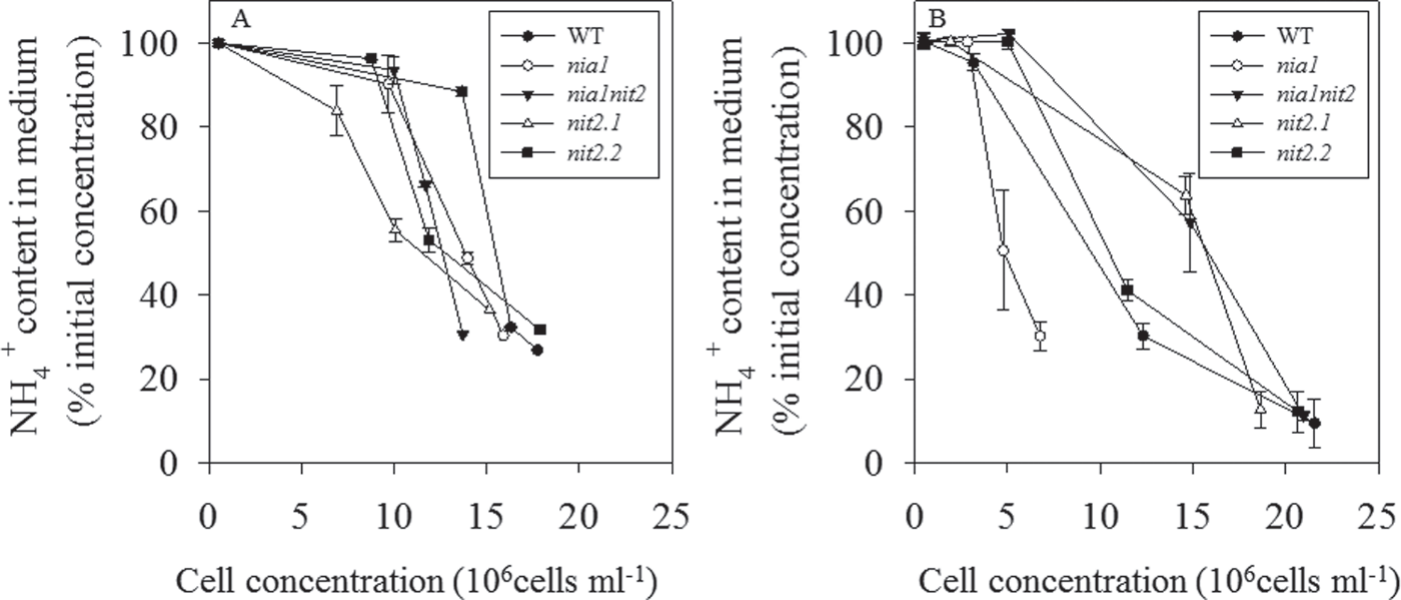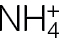 content in medium of the different strains grown on 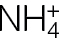 (A) and NH_4_NO_3_ (B). Cells of from wild-type and NR-deficient strains were grown in acetate medium containing 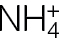 (A) or NH_4_NO_3_ (B). The initial 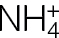 concentration in both media was 7 mmol l^–1^. Values are means±SE (*n=3*).

In order to investigate whether the differences in growth rate in the *nia1* mutant could be explained by an alteration of C and N uptake, the levels of acetate and 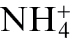 in the medium were determined during the exponential growth phase (Figs 2 and 3). Levels of 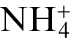 and acetate progressively decreased during exponential growth, while the concentration of algal cells increased in all mutants and the wild type under both N regimes. In either condition, extracellular acetate content reached approximately 0.6g l^–1^ in all the strains, including wild type, at the middle of the exponential phase, which corresponded to a total quantitative uptake of approximately 40% of the initial amount of acetate supplied in the medium. In all strains, about 0.2g l^–1^ of acetate remained in the medium at the end of the exponential growth phase (Figs 2 and 3). Interestingly, the *nia1* mutant showed levels of 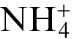 and acetate consumption similar to all other strains, while displaying a strongly reduced growth rate under NH_4_NO_3_ conditions, suggesting a stimulation of acetate and 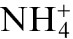 uptake in the *nia1* mutant. The impaired growth of the *nia1* strain, together with an unchanged respiratory rate under NH_4_NO_3_ fertilization (13.6±1.1 and 13.0±0.6 nmoles O_2_ min^–1^ per 10^7^ cells in wild-type and *nia1* strains, respectively), is consistent with previous data demonstrating that total respiratory rate is not affected by the source of N (Baurain *et al.*, 2003).

### Intracellular 
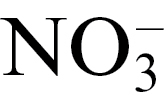
 accumulation in the *nia1* mutant under NH_4_NO_3_ nutrition

**Fig. 4.**
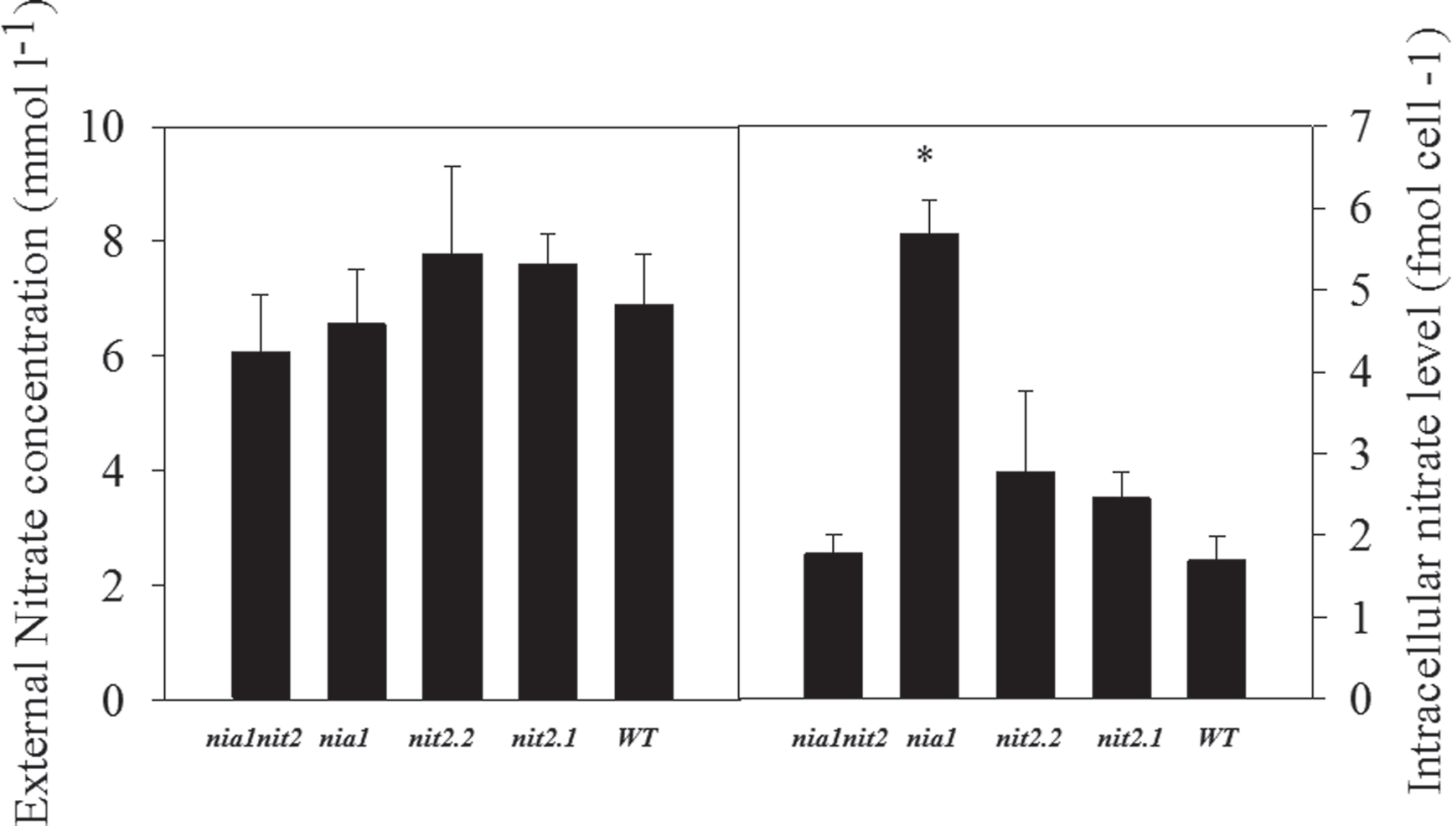
External and internal 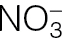 levels in wild-type and NR-deficient strains in acetate medium containing NH_4_NO_3_ as N source. Algae cultures were inoculated at 5×10^5^ cells ml^–1^. At the middle of the exponential growth phase, the external (left panel) and internal (right panel) 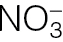 levels were determined. Values are the means±SE (*n=3*). The asterisk represents a value significantly different from that of the wild type (based on Student’s *t*-test with *P*≤0.05).

Due to the low 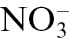 uptake in all NR-deficient lines analysed in this study (Fernandez and Cardenas, 1982), uptake was evaluated by measuring the intracellular 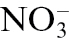 levels (Fig. 4). No intracellular 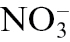 was detectable in strains grown on 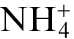 (data not shown). Under NH_4_NO_3_ nutrition, the low level of 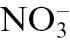 in the wild type was probably due to a decreased uptake of 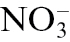 governed by 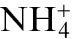 repression of 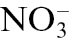 uptake and/or direct assimilation of 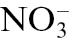. Whereas the *nit2.1*, *nit2.2*, and *nia1nit2* mutants displayed the same low amount of 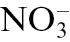 as the wild type, the *nia1* mutant line accumulated up to 3.4-fold more 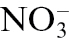 (Fig. 4). This observation is consistent with data already published, demonstrating that NIT2 is required for the expression of 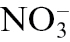 transporters (Quesada *et al.*, 1993; Camargo *et al.*, 2007).

### Induction of organic acid biosynthesis in the *nia1* mutant under NH_4_NO_3_

**Fig. 5.**
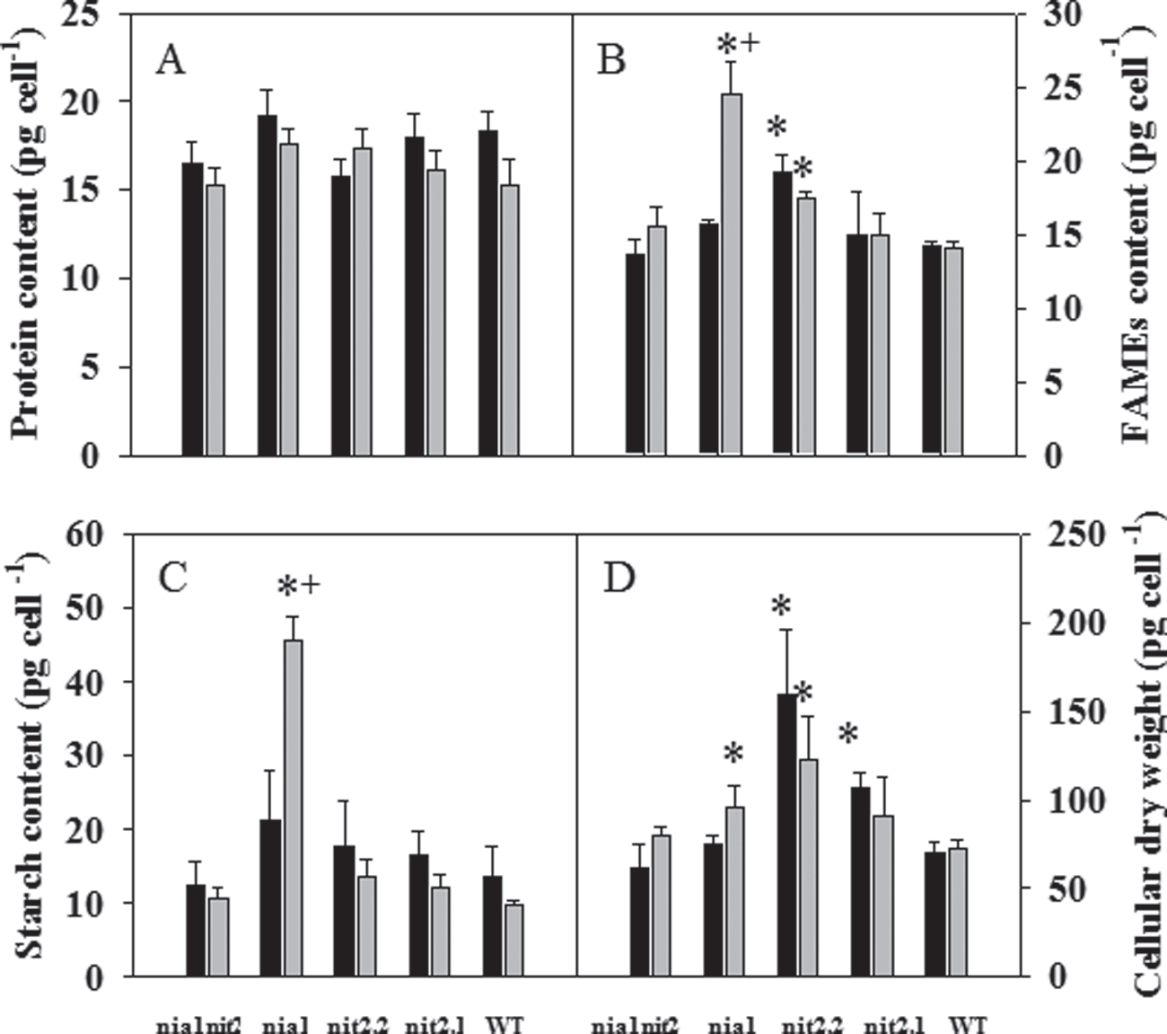
Major biomass components in wild-type and NR-deficient strains in acetate medium containing either 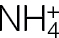 or NH_4_NO_3_ as N source. Protein content (A), total FA content (B), starch content (C) and cellular dry weight (D) were measured in mutants and wild type during the exponential growth phase under 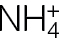 (black bars) and NH_4_NO_3_ (grey bars) conditions. Values are means±SE (*n*=3–6). Asterisks represent values significantly different from the wild type; + represents values significantly different between 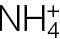 and NH_4_NO_3_ cultures for each particular strain (based on Student’s *t*-test with *P*≤0.05).

N assimilation into amino acids and proteins requires the synthesis of organic acids in the tricarboxylic acid cycle, which serve as acceptors for amino groups. The effects of both N regimes (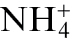 and NH_4_NO_3_) on N assimilation into amino acids were studied by measuring the total level of free amino acids per cell and malate and fumarate levels in all the different strains (Table 1). In the presence of 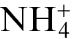, total free amino acid content was similar in NR-deficient and wild-type strains, consistent with the fact that 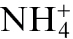 assimilation was not affected in the mutants. In the presence of NH_4_NO_3_, the *nit2.2* and *nia1* strains displayed up to 1.5–1.8-fold higher free amino acid content on a per-cell basis compared with the wild type and the *nit2.1* and *nia1nit2* mutants (Table 1). The increased 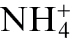 uptake and elevated free amino acid content together with an unchanged protein level in the *nia1* strain suggested either stimulation of *de novo* amino acid biosynthesis or inhibition of amino acid incorporation into the protein fraction (Fig. 5A).

**Table 1.** Cellular levels of total free amino acids, malate,and fumarate in wild-type and NR-deficient strains in both N mediaValues are the means±SE (*n=3*). Bold indicates values significantly different from the wild type; *+* represents values significantly different between 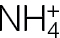 and NH_4_NO_3_ cultures on each particular strain (Student’s *t*-test with *P*≤0.05).

	Strain				
	WT	*nia1*	*nit2.2*	*nit2.1*	*nia1nit2*
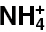 **medium**					
Total amino acids (fmol cell^–1^)	17.5±2	21.8±1.8	23.1±6	21.1±6	22.2±2.8
Malate (pmol 10^6^ cell^–1^)	280±46	**457±28**	**120±14**	**140±6**	347±34.5
Fumarate (pmol 10^6^ cell^–1^)	44.3±14	**77.4±13**	30.2±3	**15.5±5.5**	29.5±16
**NH**_**4**_ **NO**_**3**_ **medium**					
Total amino acids (fmol.cell^–1^)	11.2±1.7^**+**^	**19.8±1.2**	**17.6±1.2**	11.7±0.7^**+**^	12.4±2^**+**^
Malate (pmol 10^6^ cell^–1^)	183±19^**+**^	**992±13** ^**+**^	244±20^**+**^	207±18^**+**^	219±17^**+**^
Fumarate (pmol 10^6^ cell^–1^)	54±19	**577±23** ^**+**^	77±18^**+**^	84±6^**+**^	**108±19** ^**+**^

In 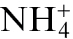 medium, the wild-type and *nia1nit2* strains contained similar levels of malate and fumarate, whereas the *nia1* mutant displayed slightly increased levels of both of these organic acids. Although the amount of malate was lower in the *nit2.1* and *nit2.2* strains than in the wild type, only the *nit2.1* strain displayed a significantly decreased fumarate level (Table 1). Compared with the wild type, only the *nia1*-deficient line accumulated up to 4-fold more malate and 10-fold more fumarate under NH_4_NO_3_ nutrition (Table 1), suggesting induction of organic acid biosynthesis for *de novo* synthesis of amino acids (Scheible *et al.*, 1997).

### Starch and triacylglycerol content are strongly affected in the *nia1* mutant under NH_4_NO_3_ nutrition

To investigate whether the changes in C/N balance in NR-deficient lines under NH_4_NO_3_ nutrition were accompanied by an alteration in C partitioning into protein, total lipid, starch levels, and cellular dry weight were determined in the different strains under both N regimes (Fig. 5A–D). The total protein fraction was approximately 18 pg per cell in the wild type and was similar to all the mutant strains under the 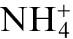 regime. The switch from 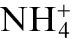 to NH_4_NO_3_ medium did not lead to a significant change in total protein level among the different strains (Fig. 5A).

**Fig. 6.**
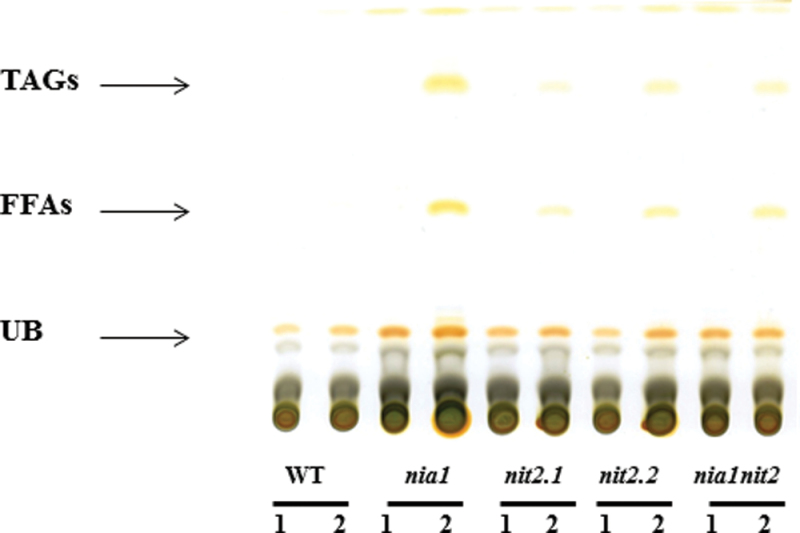
TLC analysis of neutral lipid profile of wild-type and NR-deficient strains in acetate medium containing either 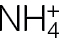 (lane 1) or NH_4_NO_3_ (lane 2) as N source. UB, unknown band.

Under N-replete conditions, most of the FAs were incorporated into polar lipids in *Chlamydomonas* cells (Fig. 6, lanes 1; Siaut *et al.*, 2011). To determine the global changes in FA biosynthesis, the total cellular lipid levels were measured by quantifying the total FAMEs using gas chromatography–mass spectrometry (GC-MS) analysis (Fig. 5B). In the presence of 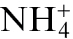 in the medium, the amounts of total FAs were similar in the wild-type, *nit2.1*, *nia1*, and *nia1nit2* strains (14±0.2, 15±3, 16±0.3, and 14±1 pg per cell, respectively), and showed a slight increase in the *nit2.2* strain (19±1.2 pg per cell). These values were consistent with data published previously (Moellering and Benning, 2010). As chain lengths and degrees of FA saturation also strongly influence the properties and quality of algae lipids, FA composition was also investigated by GC/MS analysis of FAMEs (Supplementary Tables S1 and S2 at *JXB* online).

The unchanged cellular lipid level under 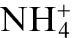 nutrition (Fig. 5B) was accompanied by a similar FA composition (Supplementary Table S1) in most of the NR-deficient strains compared with the wild type, except in the *nit2.2* mutant. Indeed the latter showed a significant 3.5-fold and 2.3-fold increase in the relative amounts of monounsaturated C16:1 (oleic acid) and C18:1 (palmitoleic acid), respectively, together with a slightly decreased proportion of polyunsaturated C18:3 (linolenic acid), suggesting a change in FA desaturation (Supplementary Table S1).

In our study, the FA composition in 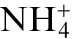 medium was similar to those published previously (EL-Sheekh, 1993; Work *et al.*, 2010). The absence of the polyunsaturated FA C16:4, could be explained by different culture conditions such as irradiance and C source.

While the total cellular FA content in wild-type, *nit2.1*, and *nit2.2* strains and the double mutant *nia1nit2* remained unchanged under both N regimes (24.4 µg per cell), the *nia1* strain showed a 1.5-fold increased total FA content under NH_4_NO_3_ compared with the 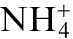 regime, corresponding to 24.4 pg per cell (Fig. 5B). Under NH_4_NO_3_, the *nia1* and *nit2.2* strains also displayed an increase in C18:2 linoleic acid (1.8- and 1.5-fold, respectively) and a slight decrease in C18:3 linolenic acid (Supplementary Table S2), suggesting inhibition of either the plastidic isoform ɷ3-desaturase FAD7 and/or the membrane-bound linoleate desaturase FAD3 located at the endoplasmic reticulum (Riekhof *et al.*, 2005).

TLC analysis of the neutral lipid profile clearly demonstrated that growth on NH_4_NO_3_, but not on 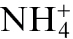, led to the accumulation of TAGs in all NR-deficient strains but not in the wild type (Fig. 6). The strongest accumulation of TAG was observed in the *nia1* strain, which also contained a generally higher FA content.

A strong interaction between starch and lipid biosynthesis pathways has been described previously in different organisms such as higher plants (Vigeolas *et al.*, 2004), *Chlorella pyrenoidosa* (Ramazanov and Ramazanov, 2006), and *Chlamydomonas* (Li *et al.*, 2010*a*,*b*; Zabawinski *et al.*, 2001). Whereas starch levels were similar in all strains under 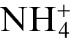 nutrition, there was a 2-fold increase in starch in the *nia1*-deficient strain compared with the wild type when grown with NH_4_NO_3_ as the source of N (Fig. 5C). In contrast, the two *nit2*-deficient strains and the *nia1nit2* double mutant displayed similar levels of this component compared with the wild type.

### Growth on NH_4_NO_3_, but not on 
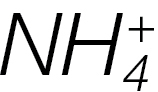
, leads to changes in biomass composition in the *nia1* mutant

Cell dry weight was also determined in all conditions in order to investigate whether the changes in storage carbohydrate contents on a per cell basis, especially for the *nia1* strain, were due to changes in C partitioning into biomass compounds or a general change in dry biomass productivity (Fig. 5D). In 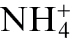 medium, wild-type, *nia1* and the double mutants showed a similar cell dry weight, while both *nit2*-deficient strains (*nit2.2*, *nit2.1*) displayed a 1.5-fold and 2.3-fold increase in dry weight per cell, respectively. In NH_4_NO_3_, all the cellular dry weights were similar to those in 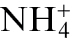 medium, indicating that the proportions of total lipids, TAGs, and starch in relation to the other biomass compounds within cells were higher in the *nia1*-deficient strain.

## Discussion

In *Chlamydomonas*, biochemical and genetic analyses have allowed the identification and characterization of most of the different components involved in 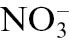 transport and assimilation, with some participating in 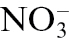 signalling pathways, including the structural gene *NIA1* encoding NR (EC. 1.6.6.2) and the *NIT2* regulator, which is considered a central regulatory gene required for 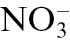 signalling. Besides 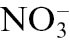, several N components have also been suggested to act as signals to regulate C and N metabolism, such as nitric oxide, glutamate, glutamine, and aspartate (Stitt and Krapp, 1999; Coruzzi and Zhou, 2001; Miller *et al.*, 2008; de Montaigu *et al.*, 2010). These data strongly suggest the presence of other potential regulatory effectors for 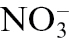 signalling, further downstream of 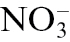 assimilation.

In this study, the effects of 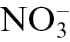 as a signalling molecule and the resulting changes in primary C metabolism were investigated in NR-deficient strains affected in either the catalytic subunit of NR (*NIA1*) or the regulatory locus (*NIT2*).

### Growth on NH_4_NO_3_, but not on 
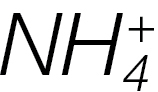
, leads to a stimulation of N and acetate assimilation into primary C metabolism in the *nia1* strain

Under NH_4_NO_3_, only the *nia1* strain displayed a growth reduction, whereas all the strains affected in the *NIT2* gene (*nit2.1*, *nit2.2*, and *nia1nit2*) displayed a similar growth pattern to the wild type (Fig. 1). This inhibition was accompanied by a stimulation of acetate uptake and an unchanged dark respiration, except that the observed growth inhibition was due to slower acetate assimilation via the respiratory chain. The stimulation of acetate uptake and assimilation in the *nia1* strain was supported by previous comparative proteomic data analysis of wild-type strains demonstrating that acetyl-coA synthases, key steps in the assimilation of acetate, are also upregulated in 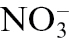-grown compared with 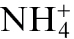
- grown cells (Gerin *et al.*, 2010).

The extracellular levels of 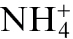 and 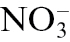 during the exponential phase were consistent with the preferential use of 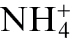 under the NH_4_NO_3_ regime. This might be due to a lower energy cost for the cells to assimilate 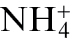 directly rather than from 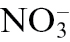 via NR, and due to the presence of a more efficient uptake and transport system (Florencio, 1983; Harris, 1989). This was also supported by a higher free amino acid content under an 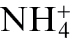 regime compared with a NH_4_NO_3_ regime in the wild type, which is probably due to a rapid incorporation of 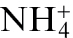 into amino acids to avoid 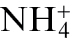 toxicity. This efficient process has been described in several organisms such as higher plants, and provides a mechanism to allow cells to cope with elevated internal free 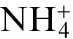 levels that would otherwise increase the intracellular pH leading to toxicity (Gerendás *et al.*, 1997).

Interestingly, compared with the wild-type and *nit2*-deficient lines displaying similar intracellular nitrate levels (2fmol per cell), the *nia1*-deficient strain accumulated up to 2.5-fold more intracellular 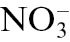.These data support the suggestion that the *NIT2* gene is involved in the control of 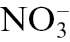 transports in the presence of intracellular 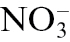 (Camargo *et al.*, 2007). The role of *NIT2* in the regulation of 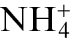 and 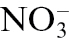 transports in the presence of intracellular 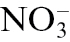 has been already described in the *nia1* strain under phototrophic conditions by transferring cells grown on 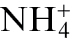 into 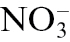 medium (Gonzalez-Ballester *et al.*, 2004; Camargo *et al.*, 2007). Based on previous studies, demonstrating that the high-affinity nitrate/nitrite transporters I, II, and III were blocked by 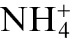, and that system IV is insensitive to 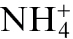, the accumulation of intracellular 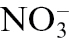 under NH_4_NO_3_ in the *nia1* strain was probably due to stimulation or induction of the transport system IV (Llamas *et al.*, 2002).

Under NH_4_NO_3_, the accumulation of organic acids and intracellular 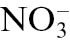 in the *nia1* strain supports the idea that both 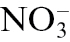 and NIT2 are involved in a signalling cascade that induces organic acid biosynthesis and initiates co-ordinated changes in C and N metabolism in *Chlamydomonas* (Zioni *et al.*, 1971; Purvis *et al.*, 1974). It has been demonstrated previously that 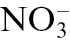 is a signal molecule in plants that has been shown to induce several thousand genes and promote diverse transcriptional responses in *Arabidopsis* (Wang *et al*., 2000, 2003).

### Under an NH_4_NO_3_ regime, starch and FA contents are strongly affected in the *nia1* mutant

Despite an increased *de novo* fatty acid synthesis suggested by a higher level of total FA content, TLC analysis of the neutral lipid fraction clearly showed that the *nia1* line displayed accumulation of TAG and free FAs, which was not observed in the wild type. Moreover, these increases were also accompanied by changes in total FA composition, such as higher C18:1/C18:3 ratios, which was observed in TAG under N starvation (Siaut *et al.*, 2011) and which is consistent with a higher TAG content. More detailed analysis of the different classes of lipids would be required to investigate the effects of lipid metabolism under NH_4_NO_3_. It is noteworthy, that all NR strains displayed a slight increased TAG and free FA content on a per-cell basis in the presence of NH_4_NO_3_, suggesting that the lack of NR itself led to changes in lipid composition. Interestingly, the increased total FA level, including TAGs, was accompanied by an accumulation of starch in the *nia1*-deficient strain (Fig. 2). The accumulation of both storage carbohydrates has already been observed in the earlier phases of N and sulfur deficiency studies in *Chlamydomonas* (Matthew *et al.*, 2009; Moellering and Benning, 2010), which is not the case in the present study. Indeed, several lines of evidence indicate that the phenotype of the *nia1* line was not due to N deprivation. First, the *nia1* strain did not turn yellow during growth, which is typical of N-starved cells (data not shown). Secondly, no evidence for a reduced 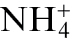 availability such as changes in protein and free amino acid levels under NH_4_NO_3_ compared with 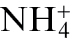 nutrition was found.

Interestingly, the *nia1* line preferentially accumulated starch rather than oil under NH_4_NO_3_. This is consistent with recent studies demonstrating that C channelling into storage lipid also occurred either when the maximal rate of starch biosynthesis was reached or blocked, or when the C source was in excess over that required for N metabolism (Work *et al.*, 2010; Fan *et al.*, 2012). The differential effect on starch and TAG synthesis could also be linked to the different energy requirements of the two biosynthetic processes. Based on theoretical considerations of the stoichiometry of the reaction pathways, addition of a six-carbon unit would cost one ATP in the case of starch and three ATPs in the case of lipid synthesis.

It is noteworthy that, while growth rates of both *nit2* mutants were similar under both N regimes, C metabolism was differentially affected in the *nit2.1* and *nit2.2* mutants under NH_4_NO_3_ nutrition. Compared with the *nit2.1* mutant, *nit2.2* contained higher levels of total FAs and free amino acids (Table 1, Fig. 5B). The stronger phenotype observed in *nit2.2* is likely to be related to two mutations in the *NIT2* gene, which are located in the third glutamine-rich region containing Ala repeats, and this domain is of crucial importance to NIT2 function (Camargo *et al.*,2007). In contrast, mutation in *nit2.1* occurs in the last exon of *NIT2* resulting in a stop codon within the RWP-RK domain. The latter has been shown to be the DNA-binding site of the homologue of the *Arabidopsis* transcription factor NLP (Konishi and Yanagisawa, 2013). The molecular nature of these two *nit2* mutants might result in a different strength of the *nit2* mutation and explain the slightly different data obtained with the two mutants.

### Contribution of starch and 
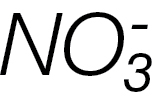
 in the control of growth

The accumulation of storage carbohydrate compounds was expected when growth is decreased, but the reasons for this growth inhibition within the *nia1* mutant remain elusive. The latter was not due to a reduction of energy processes such as respiration and photosynthesis, as dark respiration and the chlorophyll *a*/*b* ratio remained unchanged in the *nia1* strain compared with the wild type (data not shown; Kirst *et al.*, 2012).

The first possible explanation would be related to the potential effects of 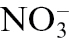 accumulation on growth in *nia1*-deficient line cells. It is commonly known that N acts as a signal to regulate and adjust growth rate in several tissues, such as roots in higher plants, and thus control C/N distribution at the whole-plant level (Stitt 1999; Wang *et al.*, 2003; Scheible *et al.*, 2004). In oilseed rape, starch metabolism has been demonstrated to be closely linked to cellular growth and differentiation (Vigeolas *et al.*, 2004; Andriotis *et al.*, 2010). Interestingly, the effects of 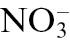 on starch biosynthesis are different from those observed in many higher plants such as tobacco and *Arabidopsis* where 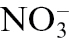 represses the expression of *AGS* gene (Scheible *et al.*, 1997), encoding the regulatory subunit of AGPase, which represents a key enzyme in starch biosynthesis.

In conclusion, our study clearly demonstrates that intracellular 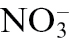 plays a major role in the regulation of starch and TAG biosynthesis in *Chlamydomonas*. This mechanism involves *NIT2* and is a *NIA1*-independent signalling pathway. Although the role of NIT2 in the 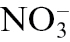 assimilation pathway is quite well documented, little is known about how internal 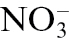 acts as signalling molecule and interacts with NIT2. Camargo *et al.* (2007) demonstrated that 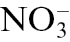 is not essential to induce *NIT2* expression, but its presence leads to the stabilization of *NIT2* transcripts. Moreover, NIT2 is composed of several different domains, characteristic of transcription factors and co-activators in other organisms, but none appears to bind 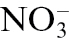. One of these is a RWP-RK, showing conservation with the *Arabidopsis* NLP7. The latter has been shown to modulate 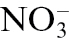 signalling and metabolism (Castaings *et al.*, 2009; Konishi and Yanagisawa, 2013). The GAF domain is in the N-terminal fragment of the protein and has been shown to bind small molecules including oxoglutarate, nitric oxide, and cGMP, but not 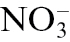. NIT2 also contains glutamine-rich domains involved in protein–protein interactions and a nuclear export sequence that binds specifically to the *NIA1* promoter regions, essential for the regulation of its expression (Camargo *et al.*, 2007). Interestingly, neither *nit2* mutant accumulated either 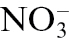 or storage compounds, indicating that this mechanism requires at least a functional RWP-RK domain and the third glutamine-rich region of the NIT2 protein.

The strong accumulation of starch and TAG in the *nia1* mutant was remarkable. To our knowledge, this is the first report of a genetic approach leading to an increase in both starch and TAG quantities of microalgae under repleted N conditions. In our point of view, due to the great economic importance and expanded use of microalgae as industrial and nutritional feedstock, this finding has obvious implications for the use of microalgae as alternative production systems for renewable energy such as biofuel. Unfortunately, its higher starch and TAG composition is also accompanied by growth inhibition. The reasons for the lower growth rate in the *nia1* mutant are still unclear and could be due to different parameters, such as the C source or external 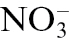 concentration.

Further studies using the double mutant *nia1sta6*, with *STAB2* encoding the small catalytic subunit of AGPase, a key step for starch biosynthesis, will be required to distinguish and clarify the contribution of starch and 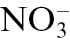 in the control of growth.

## Supplementary data

Supplementary data are available at *JXB* online.

Supplementary Table S1. Fatty acid composition of total cellular lipids from wild-type and NR-deficient strains in 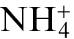 medium.

Supplementary Table S2. Fatty acid composition of total cellular lipids from wild-type and NR-deficient strains in NH_4_NO_3_ medium.

Supplementary Data

## Abbreviations:

C: carbon
FA: fatty acid
FAME: FA methyl esters
GC-MS: gas chromatography–mass spectrometry
N: nitrogen
NiR: nitrite reductase
NR: nitrate reductase
TAG: triacylglycerol
TLC: thin layer chromatography.
