# Supplementary material for: Combined intracellular nitrate and NIT2 effects on storage carbohydrate metabolism in Chlamydomonas
Source: J Exp Bot. 2013 Nov 1;65(1):23–33. doi: 10.1093/jxb/ert339 (PMC3883280; doi:10.1093/jxb/ert339)
Supplement: Supplementary Data [file supp_65_1_23__index.html]

Combined intracellular nitrate and NIT2 effects on storage carbohydrate metabolism in Chlamydomonas — Combined intracellular nitrate and NIT2 effects on storage carbohydrate metabolism in Chlamydomonas — Supplementary Data 

# Combined intracellular nitrate and NIT2 effects on storage carbohydrate metabolism in *Chlamydomonas*

## Supplementary Data

Data files

**Files in this Data Supplement:**

- Supplementary Data - Supplementary Data
