# Supplementary material for: Combined intracellular nitrate and NIT2 effects on storage carbohydrate metabolism in Chlamydomonas
Source: J Exp Bot. 2013 Nov 1;65(1):23–33. doi: 10.1093/jxb/ert339 (PMC3883280; doi:10.1093/jxb/ert339)
Supplement: Supplementary Data [file supp_ert339_jexbot105361_file001.pdf]

Combined intracellular nitrate and NIT2 effects on storage carbohydrate metabolism in *Chlamydomonas*. *Remacle C, Eppe G, Coosemans N, Fernandez E and Vigeolas H.*
Journal of Experimental Botany

SUPPLEMENTARY DATA

Supplementary table S1. Fatty acid composition of total cellular lipids from wild-type and NR deficient strains in NH_4^+ medium. *The fatty acid* composition was determined by gas chromatography Mass spectrometry of fatty acid methyl esters using log phase algae culture. Values are the means ± standard deviation (*n = 3).* Bold values represent the values significantly different from the wild type (based on Student's T- test with P ≤ 0.05).

	Strains				
	**WT**	***nia1***	***nit2.2***	***nit2.1***	***nia1nit2***
	**Fatty acid (% of total lipids)**				
Myristc acid (C14:0)	0.172±0.06	0.18±0.1	0.159±0.13	0.21±0.1	0.15±0.0
Palmitic Acid (C16:0)	28.39±2.66	29.24±1.5	24.95±4.3	27.92±4.6	27.96±1.7
Palmitoleic acid (C16:1)	3.31±0.6	2.22±1.9	**7.68±1.8**	3.04±1.2	4.468±0.4
Stearic Acid (C18:0)	9.18±0.9	8.04±1	7.67±1	7.04±0.9	8.2±0.7
Oleic Acid (C18:1,cis-9)	3.95±1.1	1.90± nd	**13.99±0.8**	**1.67±0.5**	**6.76±0.9**
Linoleic Acid (C18:2,cis-9,12)	7.44±1.3	11.57±2.3	6.69±2	10.07±1.6	7.47±0.9
Linolenic Acid (C18:3,cis-9,12,15)	43.63±2.5	42.67±4.6	**34.09±4.7**	46.80±1	40.58±2.8
Others	3.9±0.3	4.2±0.7	4.7±0.5	3.2±0.5	4.4±0.5

Supplementary table S2. Fatty acid composition of total cellular lipids from wild-type and NR deficient strains in NH_4NO_3 medium. *The fatty acid* composition was determined by gas chromatography Mass spectrometry of fatty acid methyl esters using log phase algae culture. Values are the means ± standard deviation (*n = 3)*. Bold values represent the values significantly different from the wild type (based on Student's T- test with P ≤ 0.05).

	Srains				
	**WT**	***nia1***	***nit2.2***	***nit2.1***	***nia1nit2***
	**Fatty acid (% of total lipids)**				
Myristc acid (C14:0)	nd	nd	nd	nd	nd
Palmitic Acid (C16:0)	31.22±2.46	32.98±1.63	32.56±1.24	**34.61±0.03**	31.41±2.43
Palmitoleic acid (C16:1)	2.55±0.2	2.41±0.11	**4.45±0.11**	2.23±0.03	2.77±0.41
Stearic Acid (C18:0)	4.57±0.67	3.78±0.04	4.16±0.24	4.42±0.14	4.89±0.47
Oleic Acid (C18:1,cis-9)	1.88±0.17	1.97±0.08	**5.87±0.15**	1.14±0.02	1.89±0.44
Linoleic Acid (C18:2,cis-9,12)	5.06±0.22	**9.07±0.49**	**7.42±0.51**	5.93±0.08	6.55±0.31
Linolenic Acid (C18:3,cis-9,12,15)	50.7±2.2	**45.02±0.02**	**40.43±0.01**	**47.9±0.01**	48.1±2.2
Others	4±0.41	4.71±0.35	5.06±0.29	3.81±0.07	4.35±0.23
